# Increasing venoarterial extracorporeal membrane oxygenation flow puts higher demands on left ventricular work in a porcine model of chronic heart failure

**DOI:** 10.1186/s12967-020-02250-x

**Published:** 2020-02-13

**Authors:** Pavel Hála, Mikuláš Mlček, Petr Ošťádal, Michaela Popková, David Janák, Tomáš Bouček, Stanislav Lacko, Jaroslav Kudlička, Petr Neužil, Otomar Kittnar

**Affiliations:** 1 Department of Physiology, First Faculty of Medicine, Charles University, Albertov 5, 128 00 Prague, Czech Republic; 2 Department of Cardiology, Na Homolce Hospital, Prague, Czech Republic; 3 Department of Cardiovascular Surgery, Second Faculty of Medicine, Charles University, Prague, Czech Republic; 4 Department of Cardiovascular Medicine, First Faculty of Medicine, Charles University, Prague, Czech Republic

**Keywords:** Extracorporeal membrane oxygenation, Heart failure, Swine, Hemodynamics, Heart ventricles, Artificial cardiac pacing

## Abstract

**Background:**

Venoarterial extracorporeal membrane oxygenation (VA ECMO) is widely used in the treatment of circulatory failure, but repeatedly, its negative effects on the left ventricle (LV) have been observed. The purpose of this study is to assess the influence of increasing extracorporeal blood flow (EBF) on LV performance during VA ECMO therapy of decompensated chronic heart failure.

**Methods:**

A porcine model of low-output chronic heart failure was developed by long-term fast cardiac pacing. Subsequently, under total anesthesia and artificial ventilation, VA ECMO was introduced to a total of five swine with profound signs of chronic cardiac decompensation. LV performance and organ specific parameters were recorded at different levels of EBF using a pulmonary artery catheter, a pressure–volume loop catheter positioned in the LV, and arterial flow probes on systemic arteries.

**Results:**

Tachycardia-induced cardiomyopathy led to decompensated chronic heart failure with mean cardiac output of 2.9 ± 0.4 L/min, severe LV dilation, and systemic hypoperfusion. By increasing the EBF from minimal flow to 5 L/min, we observed a gradual increase of LV peak pressure from 49 ± 15 to 73 ± 11 mmHg (P = 0.001) and an improvement in organ perfusion. On the other hand, cardiac performance parameters revealed higher demands put on LV function: LV end-diastolic pressure increased from 7 ± 2 to 15 ± 3 mmHg, end-diastolic volume increased from 189 ± 26 to 218 ± 30 mL, end-systolic volume increased from 139 ± 17 to 167 ± 15 mL (all P < 0.001), and stroke work increased from 1434 ± 941 to 1892 ± 1036 mmHg*mL (P < 0.05). LV ejection fraction and isovolumetric contractility index did not change significantly.

**Conclusions:**

In decompensated chronic heart failure, excessive VA ECMO flow increases demands and has negative effects on the workload of LV. To protect the myocardium from harm, VA ECMO flow should be adjusted with respect to not only systemic perfusion, but also to LV parameters.

## Background

Venoarterial extracorporeal membrane oxygenation (VA ECMO) represents a well-established method that can treat refractory but potentially recoverable cardiogenic shock and thus revert a dire prognosis. Temporarily, it can fully substitute the functions of the lungs and heart to maintain sufficient gas exchange and systemic blood circulation [1, 2]. VA ECMO forms a circulatory bypass by draining blood from the right atrium, passing it through the gas exchange unit, and returning the oxygenated blood into the thoracic aorta. Benefits of VA ECMO on resuscitability, improved survival, and improved neurologic outcomes have been proved both in practice [3, 4] and in animal experiments [5–7].

However, it has also been observed that VA ECMO significantly affects systemic circulation and the workload of the heart [8–13]. Increasing VA ECMO blood flow seems to negatively impact left ventricular (LV) performance; hence, it has been suggested to use only the lowest possible rate of circulatory support to protect myocardial function [8, 10]. Several causes for this negative effect have been repeatedly suspected. During high bypass flow, the inflow of blood reaches the aortic root and increases the mean aortic pressure and the LV afterload. These factors, together with preload and coronary perfusion, play key roles in heart performance. In patients with reduced myocardial contractile reserve, the increase in LV wall tension and end-diastolic pressure (EDP) due to excess afterload opposes the coronary perfusion [14] and exacerbates heart insufficiency [15]. In structural heart diseases, additional mitral regurgitation may contribute to rise in left atrial pressure; consequently, pulmonary congestion may appear as a feared complication of VA ECMO [16–18].

Especially in this context of LV overload, evaluation of VA ECMO in experiments with heart failure (HF) models have become important before translations to clinical practice. LV performance has been studied on models with intact or acutely decompensated hearts [6, 8, 19–22], but to our knowledge, there have been no experimental studies on hemodynamic effects of VA ECMO in conditions of chronic HF and its decompensation. Even though a significant part of VA ECMO clinical applications is for circulatory decompensation developed on grounds of previously present chronic heart disease, we still lack evidence from corresponding experiments. Furthermore, retrospective clinical studies have also revealed that outcome of patients treated by ECMO differs according to the “acuteness or chronicity” of cardiac disease [23].

Although there are reasons why the use of chronic HF models is scarce—time-consuming preparations, instability of heart rhythm, high mortality rates, ethical questions—the advantages of using chronic HF models are evident as they offer prolonged neurohumoral activation, general systemic adaptation, functional changes of cardiomyocytes, and structural alterations of heart valves [24–27]. In this experimental work, a chronic HF animal model was developed by long-term fast cardiac pacing which produced tachycardia-induced cardiomyopathy (TIC). TIC was first recognized in 1913 [28] and later widely used in experiments [29]. It reliably mimics decompensated dilated cardiomyopathy with low cardiac output which persists also after cessation of pacing and allows investigation of chronic HF conditions [27, 30–32].

The aim of our study was to describe hemodynamic and LV performance changes in a swine model of decompensated chronic HF, supported by gradual increase in flow of VA ECMO. We focused on LV pressure and volume changes as well as myocardial contractility and work. It is expected that VA ECMO should be able to supply enough systemic blood circulation, but the details of the negative effects on LV performance have yet to be evaluated.

## Methods

### Animal model

**Fig. 1:**
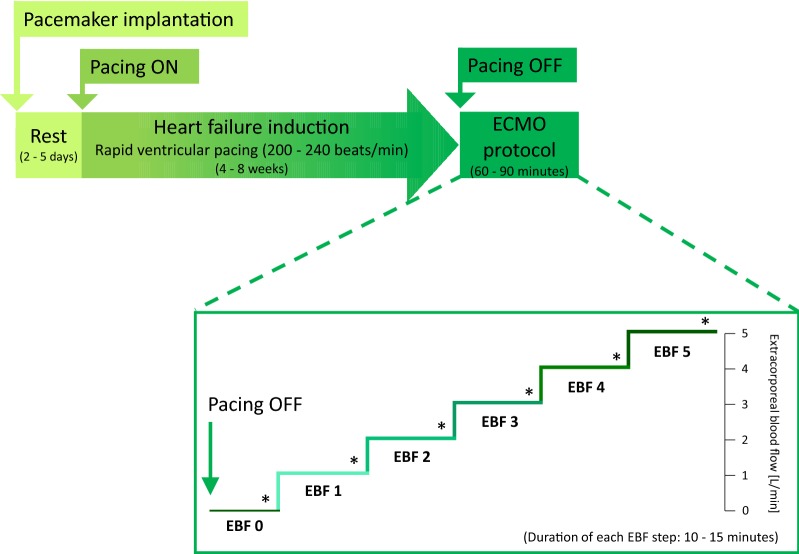
Diagram of study protocol. After pacemaker implantation, rapid ventricular pacing for 4–8 weeks led to progressive heart failure induction. When pacing was stopped, ECMO protocol consisted of stepwise increase of EBF. Data sets were collected at each EBF step (asterisk). Timeframe durations are stated in each box. *EBF* extracorporeal blood flow

According to previous studies, TIC as a form of dilated cardiomyopathy was generated by long-term rapid cardiac pacing [33–35]. Details of the methodology were recently described (Fig. 1) [32].

Five healthy crossbred female swine (*Sus scrofa* domestica) up to 6 months of age with initial weights of 37–46 kg were included in this study. After 1 day of fasting, general anesthesia was initiated with intramuscular administration of midazolam (0.3 mg/kg) and ketamine hydrochloride (15–20 mg/kg). Intravenous boluses of morphine (0.1–0.2 mg/kg) and propofol (2 mg/kg) were administered, and animals were preoxygenated and orotracheally intubated with a cuffed endotracheal tube. Total intravenous anesthesia was then continued by combination of propofol (6–12 mg/kg/h), midazolam (0.1–0.2 mg/kg/h), and morphine (0.1–0.2 mg/kg/h); all doses adjusted according to individual responses. Mechanical ventilation was provided by a Hamilton G5 closed-loop device (Hamilton Medical AG, Switzerland), set to adaptive support ventilation to maintain target end-tidal CO_2_ of 38–42 mmHg and adequate oxygen saturation of 95–99%. All procedures were performed according to standard veterinary conventions.

Under aseptic conditions and antibiotic prophylaxis (cefazolin 1 g), a single pacing lead with active fixation was inserted transvenously by fluoroscopic guidance in the apical part of right ventricle and subcutaneously tunneled to connect with an in-house modified heart pacemaker (Effecta, Biotronik SE & Co. KG, Germany), which was then implanted into a dorsal subcutaneous pocket. These arrangements proved to prevent device-related complications and allowed a wide range of high rate pacing frequencies.

Additional permanent catheter (Groshong PICC, Bard AS, USA or Arteriofix, B. Braun, Germany) was inserted through the marginal ear vein, and animals were kept in a chronic care facility under veterinary care. They were provided with free access to water and continued antibiotic regimen of cefazolin for total of 5 days.

### Pacing protocol and chronic heart failure induction

After the necessary resting period of 2–5 days, reserved for recovery from the surgical procedure, a rapid ventricular pacing was started. According to previous publications [36, 37] and our own experience [32], the pacing protocol was defined and started at a pacing rate of 200 beats/min. The frequency was then escalated to 220 beats/min after 1 week, to 240 beats/min the following week, and then sustained. Veterinary surveillance and clinical check-ups including pulse oximetry, rhythm control, and echocardiographic evaluations of myocardial contractility were performed regularly to assess the individual HF progression and pacing rate titration [25, 30]. In the case of excessive HF progression, the pacing rate was reduced for a week before increasing it again. Due to interindividual differences in response to fast pacing, time needed to produce chronic HF with profound signs of decompensation varied from 4 to 8 weeks.

At the end of pacing protocol, all animals presented consistently with symptoms of chronic HF—tachypnea, fatigue, spontaneous tachycardia of > 150 beats/min, and systolic murmurs. At further investigation, ascites, pericardial and pleural effusions, nonsustained ventricular tachycardias, dilation of all heart chambers, and significant mitral and tricuspid regurgitations were noticeable. Hemodynamics of failing circulation was denoted by arterial hypotension, and due to poor contractility and low stroke volume, cardiac output was reduced to approximately 50% of a healthy animal’s expected normal value [38]. This model of tachycardia-induced cardiomyopathy matched well to poorly compensated dilated cardiomyopathy and was preserved also after the cessation of pacing [31]. Qualities of the prepared model including neurohumoral dynamics, peripheral vascular abnormalities, and cardiac dysfunction reflected human chronic HF [26].

### Experimental preparation and hemodynamic monitoring

At this stage of decompensated chronic HF, again anesthesia and artificial ventilation were administered following principles described above, but dosing was adjusted due to low cardiac output.

Vital functions of anesthetized animals were monitored, and all invasive approaches commenced. Bilateral femoral veins and arteries, jugular vein, and left carotid artery were punctured, and intravascular accesses ensured by standard percutaneous intraluminal sheaths. Right carotid and subclavian arteries were surgically exposed, and circumjacent ultrasound flow probes of appropriate sizes attached (3PSB, 4PSB, or 6PSB, Scisense, Transonic Systems, USA), enabling continuous detection of blood flow velocities. Near-infrared spectroscopy (INVOS Oximeter, Somanetics, USA) with sensors placed on forehead and right forelimb was used to monitor cranial and peripheral regional tissue oxygen saturations (rSO_2_).

Intravenous anticoagulation was initiated by unfractionated heparin bolus (100 IU/kg IV), followed by continual infusion, maintaining an activated clotting time (ACT) of 200–300 s (Hemochron Junior + , International Technidyne Corporation, USA), and a set of invasive monitoring equipment was introduced. A balloon Swan-Ganz catheter was placed through femoral vein to the pulmonary artery allowing thermodilution-derived continuous cardiac output, mixed venous oxygen saturation (SvO_2_), pulmonary artery, and pulmonary wedge pressure assessments (CCO Combo Catheter; Vigilance II, Edwards Lifesciences, USA). Through the aortic valve, a pressure–volume catheter (7F VSL Pigtail, Scisense, Transonic Systems, USA) was introduced and positioned in the LV cavity. Central venous pressure (CVP) was measured via jugular vein and arterial pressure in femoral artery using fluid-filled pressure transducers (TruWave, Edwards Lifesciences, USA).

Intracardiac and transthoracic echocardiography probes (AcuNav IPX8, Acuson P5-1 and X300 ultrasound system, Siemens, USA) were used for 2D and color Doppler imaging. ECG, heart rate (HR), pulse oximetry, capnometry, rectal temperature, and SvO_2_ were measured continuously; blood gas parameters were evaluated by a bedside analysis system (AVL Compact 3, Roche Diagnostics, Germany).

### Left ventricular parameters and stroke work analysis, hemodynamic parameters

**Fig. 2:**
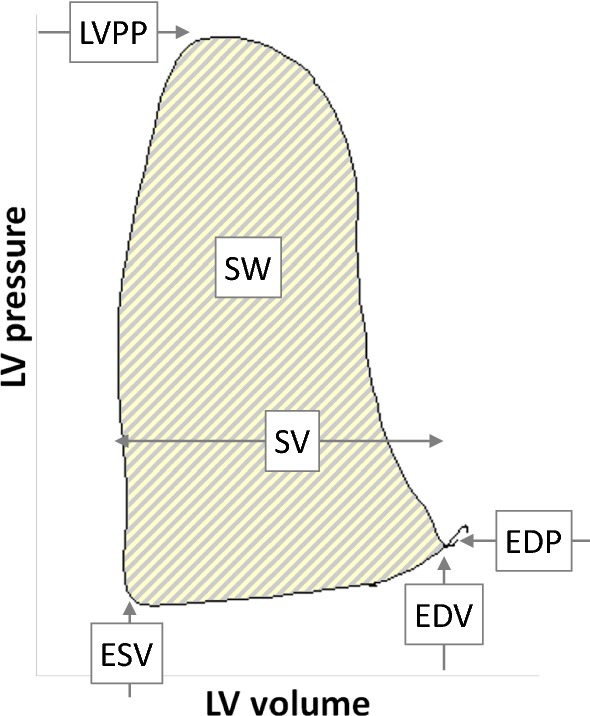
Pressure–volume loop diagram example recorded by PV catheter. A single cardiac cycle is defined by left ventricular (LV) dimensions, pressures, and work. *ESV* end-systolic volume, *EDV* end-diastolic volume, *EDP* end-diastolic pressure, *LVPP* LV peak pressure, *SV* stroke volume, *SW* LV stroke work

To register instant volume and pressure in the LV chamber, a pressure–volume (PV) conductance catheter was passed via left carotid arterial approach, retrogradely through the aortic valve into the LV. Its fluoroscopy and echocardiography guided position was set stable before the protocol started to obtain optimal PV loop morphology (Fig. 2). Volume measurements were calibrated by thermodilution-derived cardiac output at baseline.

Measured LV parameters included end-diastolic pressure and volume (EDP and EDV), end-systolic volume (ESV), LV peak pressure (LVPP), stroke work (SW; defined as LV pressure integral with respect to volume), and maximal positive change of LV pressure, defined as first time derivative of LV pressure (dP/dt_max_). When normalized to EDV, dP/dt_max_/EDV represents a preload independent index of LV contractility [39–43].

Additional parameters were stroke volume (SV), left ventricular ejection fraction (EF), mean arterial flows in carotid and subclavian arteries, and their pulsatility indices.

### ECMO

**Fig. 3:**
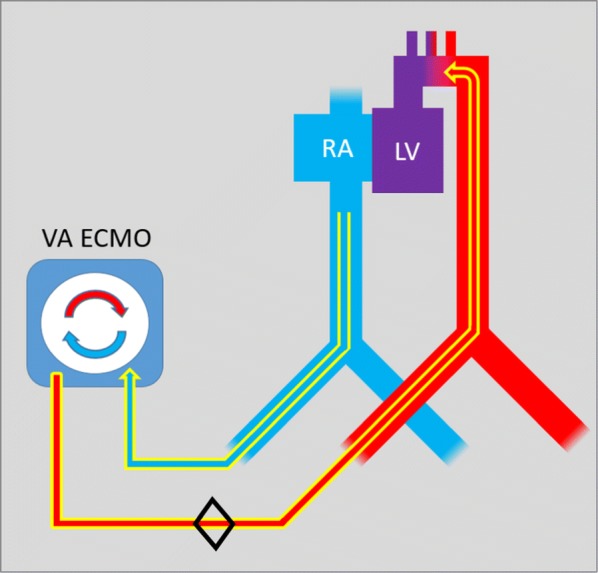
Femoro-femoral VA ECMO scheme. Venous blood is drawn by inflow cannula from right atrium (RA). Then it continuous through the gas exchange unit by the force of centrifugal pump and oxygenated is returned to the descending part of thoracic aorta. *LV* left ventricle. Black diamond showing the placement of EBF flow probe

After intravenous systemic heparinization, extracorporeal circulation was maintained by a femoral VA ECMO system compounded of Levitronix Centrimag console (Thoratec, USA) with a centrifugal pump, hollow fiber microporous membrane oxygenator (QUADROX-i Adult, Maquet Cardiopulmonary, Germany), and tubing set with two percutaneous cannulas (Medtronic, USA) which were introduced by the Seldinger technique through punctures of the unilateral femoral vein and artery (Fig. 3). The tip of venous inlet cannula (23 Fr) was advanced to the right atrium, and the tip of arterial outlet cannula (18 Fr) reached the thoracic descending aorta, with both positions verified by fluoroscopy. Fully assembled ECMO circuit [44] was primed with saline solution, and extracorporeal blood flow (EBF) was initiated at flow rate of 300 mL/min to prevent thrombus formation inside ECMO circuit while having a neglectable impact on the systemic circulation. EBF was registered by a separate circumjacent flow probe (ME 9PXL, Transonic Systems, USA) attached to the ECMO outlet cannula.

Blood gas analysis was checked continuously (CDI Blood Parameter Monitoring System 500, Terumo Cardiovascular Systems Corporation, USA) throughout the whole experiment; the fraction of oxygen and air flow through the oxygenator were adjusted to maintain pO_2_ 100–120 mmHg, pCO_2_ 35–45 mmHg, and pH 7.35–7.45 in the blood leaving the oxygenator.

### Experimental protocol and data acquisition

After instrumentation was completed and ventricular pacing discontinued, all animals were studied in sinus rhythm. Mechanical ventilation was adjusted to keep oxygen saturation above 95% and end-tidal CO_2_ within range 38–42 mmHg. Crystalloid infusion was continuously administered (2.5–5.0 mL/kg/h) to reach and maintain a mean CVP at least 5 mmHg. ACT was kept between 200 and 300 s by intravenous heparin administration, and normothermia was maintained. No inotropic agents were used during the protocol.

Under conditions of profound chronic HF, the ECMO protocol was initiated. By changing the ECMO pump rotation speed, the EBF was set according to a standardized ramp protocol (Fig. 1). EBF was gradually increased by increments of 1 L/min every 10–15 min from minimal flow to 5 L/min, and these stepwise categories with constant EBF were referred to as EBF 0, 1, 2, 3, 4, and 5. At each step, animals were allowed to stabilize to a steady state condition in which parameters including PV loop data were recorded. Sets of data were then averaged from three end-expiratory time points. If present, premature beats were omitted from the analyses. At the completion of each study, the animals were euthanized, and a necropsy performed to look for potential cardiac anomalies.

### Statistical analysis

All data sets were tested for normality and are expressed as mean ± standard error of mean (SEM). Comparisons between different levels of EBF were analyzed—by using the Friedman test with Dunn’s multiple comparison and linear regression with Pearson correlation and scatter plots were used for comparisons in between models. A two-sided P-value < 0.05 was considered statistically significant.

Recordings were sampled at 400 Hz by PowerLab A/D converter and continuously recorded to LabChart Pro Software (ADInstruments, Australia). Statistical analyses and graphical interpretations were performed in Prism 6 (GraphPad, USA) and Excel (Microsoft, USA).

## Results

**Fig. 4:**
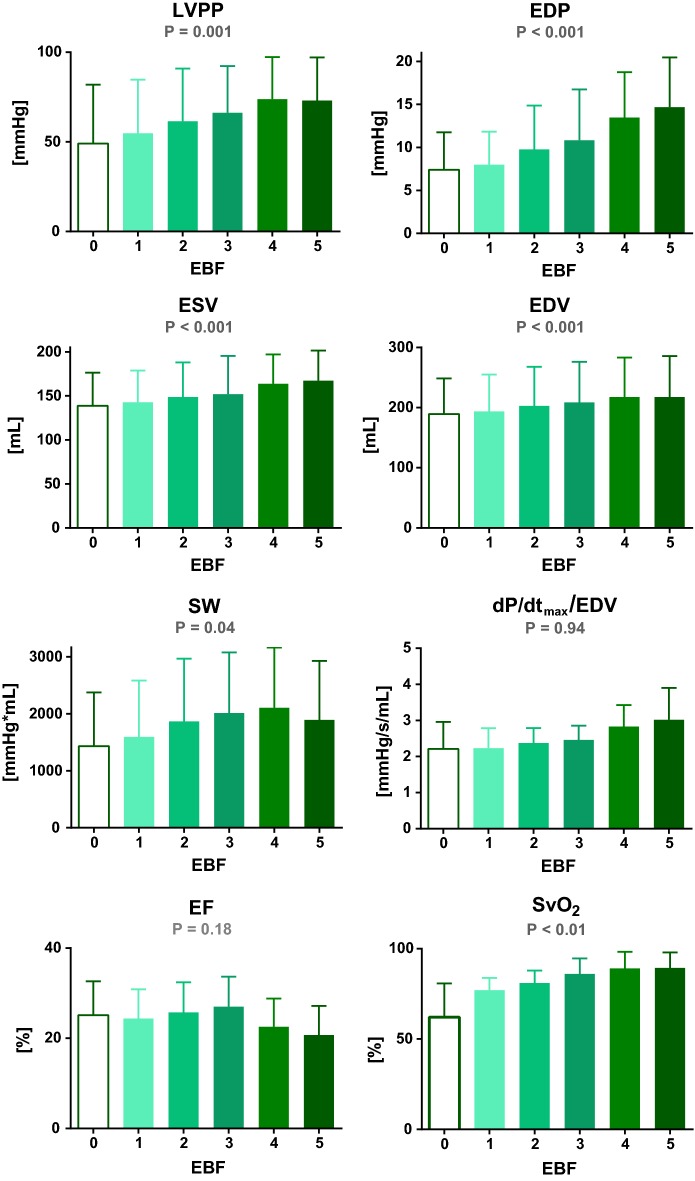
Effects of venoarterial extracorporeal membrane oxygenation blood flow (EBF in L/min) on selected hemodynamic parameters in a porcine model of chronic heart failure. *LV* left ventricle, *LVPP* LV peak pressure, *EDP* LV end-diastolic pressure, *ESV* LV end-systolic volume, *EDV* LV end-diastolic volume, *SW* LV stroke work, *dP/dt*_*max*_*/EDV* maximal positive LV pressure change normalized to EDV, *EF* LV ejection fraction, *SvO*_*2*_ mixed venous oxygen saturation

Detailed results are summarized in Table 1 and Fig. 4. In the all animals in our study, fast ventricular pacing for 4–8 weeks generated TIC with signs of decompensated HF which was denoted by baseline values of cardiac output 2.9 ± 0.4 L/min at rest, severe dilation of all heart chambers, valves insufficiency, and systemic hypotension. Left ventricular EF evaluated by echocardiography was below 30% in all animals; initial mean heart rate of sinus rhythm was 100 ± 19 beats/min; dyssynchrony of LV contraction was obvious. Baseline SvO_2_ value of 62 ± 8% at rest corresponded with inadequate tissue oxygen delivery in our model, and elevated CVP underlined congestion.

**Table 1:** Hemodynamic and pressure–volume characteristics

Parameter	Units	VA ECMO blood flow						P	Relative change
		EBF 0	EBF 1	EBF 2	EBF 3	EBF 4	EBF 5		EBF 0–5 (%)
Ventricular hemodynamics									
LVPP	mmHg	49 ± 5	55 ± 13	61 ± 13	66 ± 12	74 ± 10*	73 ± 11*	0.001	49
EDP	mmHg	7 ± 2	8 ± 2	10 ± 2	11 ± 3	13 ± 2*	15 ± 3*	< 0.001	114
ESV	mL	139 ± 17	143 ± 16	148 ± 18	152 ± 19	164 ± 15*	167 ± 15*	< 0.001	20
EDV	mL	189 ± 26	194 ± 27	203 ± 29	209 ± 30	217 ± 29*	218 ± 30*	< 0.001	15
SV	mL	51 ± 20	51 ± 20	56 ± 20	59 ± 20	55 ± 21	52 ± 21	0.03	2
EF	%	25 ± 7	24 ± 6	26 ± 7	27 ± 7	23 ± 6	21 ± 6	0.18	− 16
HR	beats/min	101 ± 22	96 ± 19	93 ± 17	90 ± 13	90 ± 14	86 ± 14	0.34	− 15
SW	mmHg*mL	1434 ± 941	1595 ± 987	1867 ± 1102	2014 ± 1062	2105 ± 1060*	1892 ± 1036	0.04	32
dP/dt_max_/EDV	mmHg/s/mL	2.2 ± 0.8	2.2 ± 0.6	2.4 ± 0.4	2.5 ± 0.4	2.8 ± 0.6	3 ± 0.9	0.94	36
Perfusion parameters									
Carotid flow	mL/min	211 ± 72	291 ± 62	314 ± 57	356 ± 57	447 ± 64*	479 ± 58*	< 0.001	127
Subclavian flow	mL/min	103 ± 49	128 ± 44	158 ± 40	208 ± 47	266 ± 47*	296 ± 54*	< 0.001	187
Cranial rSO_2_	%	57 ± 6	60 ± 4	67 ± 5	69 ± 5	72 ± 4*	74 ± 3*	< 0.001	30
Forelimb rSO_2_	%	37 ± 6	46 ± 5	58 ± 5	67 ± 6	72 ± 7*	77 ± 6*	< 0.001	108
SvO_2_	%	62 ± 8	77 ± 3	81 ± 3	86 ± 4	89 ± 4*	89 ± 4*	< 0.001	44
CVP	mmHg	14 ± 2	11 ± 2	10 ± 2	8 ± 2*	9 ± 2*	8 ± 2*	0.001	− 43

For each step of increasing extracorporeal blood flow (EBF in L/min), hemodynamic values are expressed as mean ± SEM

*LV* left ventricle, *LVPP* LV peak pressure, *EDP* LV end-diastolic pressure, *ESV* LV end-systolic volume, *EDV* LV end-diastolic volume, *SV* stroke volume, *EF* LV ejection fraction, *HR* heart rate, *SW* LV Stroke work, *dP/dt*_*max*_*/EDV* maximal time derivative of LV pressure change normalized to EDV, *SvO*_*2*_ mixed venous oxygen saturation, *CVP* central venous pressure

Values significantly different from EBF 0 are marked with *

After connecting VA ECMO, stepwise increments of EBF from minimal to maximal flow led to gradual and dramatic changes in LV hemodynamic parameters. LVPP increased by 49% from 49 ± 15 mmHg to 55 ± 13, 61 ± 13, 66 ± 12, 74 ± 10, and 73 ± 11 mmHg (for EBF 0 to 5, P = 0.001), and EDP increased by 114% from 7 ± 2 mmHg to 8 ± 2, 10 ± 2, 11 ± 3, 13 ± 2, and 15 ± 3 mmHg (for EBF 0 to 5, P < 0.001). Every escalation of EBF emphasized LV dilation. ESV increased severely by 20% from 139 ± 17 mL to 143 ± 16, 148 ± 18, 152 ± 19, 164 ± 15, and 167 ± 15 mL and EDV by 15% from 189 ± 26 mL to 194 ± 27, 203 ± 29, 209 ± 30, 217 ± 29, and 218 ± 30 mL (both for EBF 0 to 5, P < 0.001).

On the other hand, SV and EF changed only with less significant mean differences and both reached highest values at EBF 3 L/min. In detail, SV changed from 51 ± 20 mL to 51 ± 20, 56 ± 20, 59 ± 20, 55 ± 21, and 52 ± 21 mL (P = 0.03), and EF from 25 ± 7% to 24 ± 6, 26 ± 7, 27 ± 7, 23 ± 6, and 21 ± 6% (P = 0.18). Mean HR tended to decline with every increase in EBF—from 101 ± 22 beats/min to 96 ± 19, 93 ± 17, 90 ± 13, 90 ± 14, and 86 ± 14 beats/min (for EBF 0 to 5, P = 0.34).

Left ventricular SW was calculated from measured pressure–volume loops and exhibited significant flow-dependent increases from 1434 ± 941 mmHg$*$mL to 1595 ± 987, 1867 ± 1102, 2014 ± 1062, 2105 ± 1060, and 1892 ± 1036 mmHg$*$mL (EBF 0 to 5, P = 0.04). However, preload independent index of LV contractility represented by dP/dt_max_/EDV ratio showed no consistent trend during the ECMO protocol—from 2.2 ± 0.8 mmHg/s/mL to 2.2 ± 0.6, 2.4 ± 0.4, 2.5 ± 0.4, 2.8 ± 0.6, and 3.0 ± 0.9 mmHg/s/mL (EBF 0 to 5, P = 0.94).

Arterial blood flow increased with every increase of EBF—in total by 127% in the carotid and by 187% in the subclavian artery, while pulsatility indices dropped significantly. SvO_2_ increased to 77 ± 3% with EBF 1 and reached > 80% with all higher EBF steps (P < 0.001). Similarly, rSO_2_ values were low at baseline but increased promptly with ECMO flow. With increasing EBF, the average value of CVP did gradually fall, but not under 7 mmHg, avoiding ECMO underfilling (P = 0.001).

Postmortem autopsies did not reveal any shunt or other cardiac anomaly, but myocardial hypertrophy (heart weight 471 ± 127 g).

## Discussion

VA ECMO is being used as an ultimate method in cases of severe circulatory decompensation, but multiple clinical and experimental studies have documented adverse changes in LV function with the increased EBF [2, 8, 12, 18] for both cardiac [13] and respiratory [45] compromised patients. The incidences of these complications vary widely between 12 and 68% [13, 46–48] and are still believed to be underreported [13, 46].

The goal of our experiment was to assess the response of hemodynamic parameters and LV workload to different levels of EBF. We chose a porcine model of TIC, developed by long-term fast ventricular pacing, over weeks resulting in symptoms and signs of anatomical, functional, and neurohumoral profiles similar to human chronic HF. As far as we are informed, this study is unique in that it describes the hemodynamic effects of VA ECMO in decompensated chronic HF model. An important feature of our protocol is also adequate lung ventilation which ensures that coronary arteries receive well-oxygenated blood during all rates of EBF. Therefore, reported changes in LV hemodynamics should not be attributed to coronary ischemia as a result of poor oxygenation.

With initiation of the extracorporeal circulation and stepwise increase of EBF, progressive dilation of LV was observed. Affected were both the end-diastolic (in total by 15% from EBF 0 to 5) and even more, the end-systolic volume (by 20%). This increase in LV dimensions was strongly pronounced between EBF 0 and EBF 4; by increasing the flow beyond this point, left ventricle did not significantly further dilate.

**Fig. 5:**
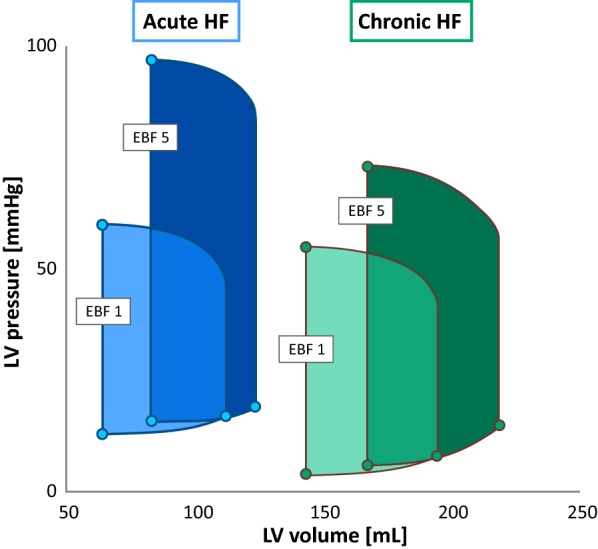
Effect of increasing EBF on acute (blue) and chronic (green) heart failure. Averaged PV loops for EBF 1 and EBF 5 are demonstrating the change of LV parameters by increasing EBF. Acute heart failure data used from Ostadal et al. study [8]. *EBF* ECMO blood flow

**Fig. 6:**
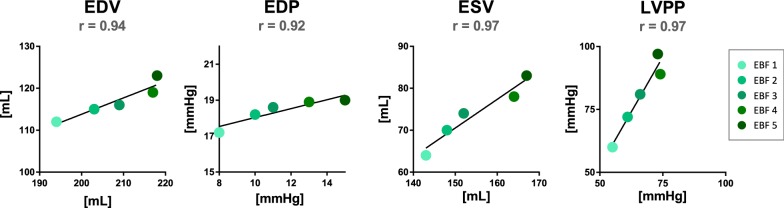
Scatter plots of LV pressure–volume parameters showing effects of increasing EBF on chronic (horizontal axes) and on acute heart failure (vertical axes). Acute heart failure data used from Ostadal et al. 2015 study [8]. In each graph, both axis (horizontal and vertical) presented with identical scales. For all, P < 0.05

LV pressures demonstrated upward trends as well. LVPP increased by 49% but still remained abnormally low despite high EBF. LV EDP was initially elevated to 7 mmHg due to abnormal LV filling dynamics, but when compared to a model of acute heart failure [8], the EDP elevation during low EBF in TIC was pointedly lower (Figs. 5 and 6). This may be explained by the chronicity of our HF model which led to massive ventricular dilation without appreciable thickening of its wall, higher ventricular compliance, and thus less pronounced EDP elevation. With ECMO flows from EBF 0 to 5, EDP increased significantly further by over 114% leading to high preload and high end-diastolic wall tension, possibly opposing coronary perfusion [14].

On the other hand, SV and EF had a different course when increasing EBF. Both these measures of LV ejection showed increase from EBF 1 to EBF 3, but with higher EBF, their mean values decreased. Systolic ejection trended to maximum at middle levels and to minimum at the highest levels of VA ECMO support. Such a trend was not observed in acute cardiogenic shock model induced by regional myocardial hypoxemia where ejection continued to decline [8] but was observed in another model of global myocardial hypoxia supported by stepwise VA ECMO [49]. Interestingly, in the latter work, this trend was observed regardless of pulsatile or non-pulsatile ECMO flows.

SW, defined as the area of PV loop and representing the instant LV workload, was expected to be proportional to ventricular systolic pressure and SV [40, 41]. In our measurements, demands on the LV work measured by SW showed significant increase by 40% from EBF 0 to EBF 4, and with further escalation of ECMO flow to EBF 5, declined. In fact, SW is well respecting the trend of LVPP and SV.

The maximal positive LV pressure change is considered to be one of isovolumetric phase contractility indices. Due to severe LV dilation in our experiment, we used this index normalized to EDV, dP/dt_max_/EDV, as recommended for its high sensitivity for global inotropic state, irrespective of ventricular loading conditions [39, 41, 43]. It has been confirmed that its linear relation is affected less by afterload compared to the end-systolic PV ratio [43]. As expected, the initial value in our TIC model was significantly below normal. Further across the stepwise protocol of EBF, the ratio of dP/dt_max_/EDV continued to increase but did not meet statistical significance. For isovolumetric phase of contraction, this could imply that LV did not lose its ability to contract. In individuals where the dP/dt_max_/EDV ratio did not decrease with higher EBF, this could be explained by unrestricted or at least, sufficient coronary perfusion and secondarily by preserved potential of contractility. A similarly designed study reported hemodynamic effects of VA ECMO, but applied to healthy canine circulation [14]. Although they reported reduction of coronary flow with increasing EBF, myocardial oxygen consumption was not reduced. In a similar sense, our data did not indicate critically inadequate myocardial perfusion on any of EBF levels.

Blood propelled to the aortic root, especially potentiated by aortic and mitral regurgitations, often leads to increased left atrial pressure [15]. Yet, in none of our cases, progression into pulmonary edema was observed, which could be due to the short-term duration of extracorporeal support and sufficient right heart unloading.

The combination of increasing SW and not declining dP/dt_max_/EDV ratio demonstrate increased demands on LV work, placed by the high afterload, and this increase in LV work occurs concurrently with LV dilation [41].

As the metabolic rate and hematocrit remain unchanged, systemic organ perfusion can be predicted by arterial flows and mixed venous blood saturation. We previously reported linear correlation of tissue saturation and regional arterial flow in VA ECMO support of chronic HF. Both tissue saturation and regional arterial flow demonstrated significant increases with respect to EBF [7]. Low initial value of SvO_2_ improved already at level of EBF 1, suggesting sufficient systemic perfusion with only minor extracorporeal support. Not surprisingly, with every higher EBF step, average SvO_2_ and carotid arterial flow rose, but the pulsatility index gradually decreased, demonstrating loss of aortic pulse pressure and the dominance of ECMO blood flow in systemic circulation [5, 7]. Practically, the value of regional tissue saturation together with SvO_2_ have the potential to serve as additional methods to secure vital organ perfusion in ECMO therapy guidance.

A recent study by our group described hemodynamic effects of VA ECMO on an innovative animal model of acute HF induced by hypoxic coronary perfusion [8, 20], and these findings were later confirmed by computer modeling [9]. Similar to our current design, the effects of increasing EBF on LV were reported. Figure 5 is depicting PV loops of acute and chronic model reactions to VA ECMO flow which can offer insights into hemodynamic changes in both models.

In comparison to our current study, baseline ESV and EDV in acute HF were reported to be of far smaller dimensions (46% for ESV, 58% for EDV) and the effect of faster EBF was different in acute HF—by increasing EBF, ESV increased by 31% (64 ± 11 mL to 83 ± 14 mL, P < 0.001), but EDV only by 10% (112 ± 19 mL to 123 ± 20 mL, P = 0.43, both for EBF 1 to 5). Likewise, increase of LVPP was more pronounced (by 67%; from 60 ± 7 to 97 ± 8, P < 0.001), but changes of EDP were only mild (17.2 ± 1.4 to 19.0 ± 2.9, P = 0.87, both for EBF 1 to 5). In both experiments, SW followed the same trend, reaching the highest value at EBF 4, and HR declined with every increment of EBF.

In Fig. 6, PV characteristics of this acute model are plotted against our results. Values of EDV, ESV, EDP, and LVPP for corresponding EBF steps reveal linear relations with dissimilar slopes (r = 0.94, r = 0.97, r = 0.92, and r = 0.97, respectively, all P < 0.05).

In another model of acute HF generated by hypoxic myocardial perfusion, Shen et al. reported a decline of dP/dt_max_ and LVPP associated with VA ECMO flow, but in their settings all of the coronary vascular bed received hypoxemic blood [22].

When summarized, in our chronic HF model, EDP was lower at baseline but increased more with higher EBF, and end-diastolic dilation was more pronounced. EF and SV were proved to decline only in the case of acute hypoxic HF. One possible explanation is that in our protocol, high ECMO flow improves the coronary perfusion with oxygen rich blood, in contrast to the acute models, where a major portion of LV myocardium is perfused by constant flow of hypoxic blood, stunned, and therefore cannot keep pace and eject against increasing afterload. Long-term adaptation to chronic HF conditions with humoral activation during TIC model induction could be another possible explanation.

Although the impact of LV overload on clinical outcomes is mostly unknown and the literary evidence limited, it is expected that it negatively affects patients’ recovery [13, 50] and in minority of patients, the status can further progress into pulmonary congestion or edema [13, 17, 18, 50]. In addition, recent small clinical and experimental series have shown that the severity of LV overload and distention correlates well with the rate of EBF [8, 11, 51].

In clinical settings, echocardiography can serve as a method of choice to evaluate the ventricular response to volume loading during or while weaning from ECMO [11, 50, 51]. Assessments of SV and filling pressures, together with pulmonary wedge pressure, lung fluid accumulation, and regional saturations, are available measures which should be operated on daily basis [13, 50, 52]. Having these in hands, partial flows of extracorporeal support in conjunction with aggressive inotropic support have been suggested in effort to prevent LV overload [7, 8, 13].

In the presented study we showed how VA ECMO increases the demands on LV work and that despite significant increase of EDP, negative effect on load-independent contractility was not observed, suggesting sufficient myocardial oxygenated blood supply. Several methods have been proposed to unload the ventricles under VA ECMO therapy but are associated with increased rate of complications and the clinical evidence is limited to few single center small cohorts and case reports [13, 16–18, 50]. In a recent report, 15 out of 36 patients with LV distention on VA ECMO required some venting strategy to reduce wall stress [13]. It has also been shown that even a small venting catheter of 7 or 8 Fr in left atrium [47] or LV [16] seems sufficient and that its early applications result in lower mortality [18, 48]. However, the clinical relevance of these effects still remains uncertain.

We assume that to decrease the risk of LV overload, VA ECMO flow should be maintained at the lowest level securing adequate tissue perfusion.

Even though the study protocol was prepared carefully and the total number of studied animals was adequately reasonable, several limitations must be considered. First, real isovolumetric phase was absent on recorded PV loops, as it could only be seen in cases with intact heart valves. In real measurements, LV volume may never stay constant due to valve insufficiency. The aortic regurgitation in our experiments was caused by PV loop catheter and was not considered severe on echocardiography. Second, contractility indices are highly sensitive but are also known to have a low reproducibility for contractile status. Therefore, myocardial perfusion cannot be directly deducted. In future studies, coronary arterial flow or oxygen consumption should be assessed. A third limitation could be seen in the relatively brief duration of extracorporeal support used in this study compared to common durations of ECMO therapy. To test long-term effects, experiments should be extended, and lung fluid content assessed. Lastly, right ventricular workload should also be considered.

## Conclusion

We confirm that increase in EBF during VA ECMO therapy increases demands on LV performance, and our work extends this observation to conditions of decompensated chronic HF. We conclude that to protect the myocardium from harm, EBF of VA ECMO should be set with respect to not only systemic perfusion but also to LV parameters.

## Data Availability

The datasets used and analyzed during the current study are available from the corresponding author on request. In “Discussion” section and Figs. 5 and 6 we reference to data from Ostadal et al. study [8] (10.1186/s12967-015-0634-6) with generous permission of the author.

## Abbreviations

ACT: Activated clotting time
CVP: Central venous blood pressure
EBF: Extracorporeal blood flow
(VA) ECMO: (Venoarterial) extracorporeal membrane oxygenation
EDP: LV end-diastolic pressure
EDV: LV end-diastolic volume
EF: LV ejection fraction
ESV: LV end-systolic volume
HF: Heart failure
HR: Heart rate
LV: Left ventricle
LVPP: LV peak pressure
PV (loop): Pressure–volume (loop)
dP/dt_max_: Maximal positive LV pressure change
dP/dt_max_/EDV: dP/dt_max_ normalized to EDV
rSO_2_: Regional tissue saturation
SV: LV stroke volume
SvO_2_: Mixed venous oxygen saturation
SW: LV stroke work
TIC: Tachycardia-induced cardiomyopathy

## References

[1] Abrams D, Combes A, Brodie D (2014). Extracorporeal membrane oxygenation in cardiopulmonary disease in adults. J Am Coll Cardiol.

[2] Brogan TV, Lequier L, Lorusso R, MacLaren G, Peek G (2017). Extracorporeal life support: the ELSO red book.

[3] Combes A, Leprince P, Luyt CE, Bonnet N, Trouillet JL, Leger P, Pavie A, Chastre J (2008). Outcomes and long-term quality-of-life of patients supported by extracorporeal membrane oxygenation for refractory cardiogenic shock. Crit Care Med.

[4] Pranikoff T, Hirschl RB, Steimle CN, Anderson HL, Bartlett RH (1994). Efficacy of extracorporeal life support in the setting of adult cardiorespiratory failure. ASAIO J.

[5] Belohlavek J, Mlcek M, Huptych M, Svoboda T, Havranek S, Ost’adal P, Boucek T, Kovarnik T, Mlejnsky F, Mrazek V, Belohlavek M, Aschermann M, Linhart A, Kittnar O (2012). Coronary versus carotid blood flow and coronary perfusion pressure in a pig model of prolonged cardiac arrest treated by different modes of venoarterial ECMO and intraaortic balloon counterpulsation. Crit Care.

[6] Mlcek M, Ostadal P, Belohlavek J, Havranek S, Hrachovina M, Huptych M, Hala P, Hrachovina V, Neuzil P, Kittnar O (2012). Hemodynamic and metabolic parameters during prolonged cardiac arrest and reperfusion by extracorporeal circulation. Physiol Res.

[7] Hala P, Mlcek M, Ostadal P, Janak D, Popkova M, Boucek T, Lacko S, Kudlicka J, Neuzil P, Kittnar O (2016). Regional tissue oximetry reflects changes in arterial flow in porcine chronic heart failure treated with venoarterial extracorporeal membrane oxygenation. Physiol Res.

[8] Ostadal P, Mlcek M, Kruger A, Hala P, Lacko S, Mates M, Vondrakova D, Svoboda T, Hrachovina M, Janotka M, Psotova H, Strunina S, Kittnar O, Neuzil P (2015). Increasing venoarterial extracorporeal membrane oxygenation flow negatively affects left ventricular performance in a porcine model of cardiogenic shock. J Transl Med.

[9] Broome M, Donker DW (2016). Individualized real-time clinical decision support to monitor cardiac loading during venoarterial ECMO. J Transl Med.

[10] Seo T, Ito T, Iio K, Kato J, Takagi H (1991). Experimental study on the hemodynamic effects of veno-arterial extracorporeal membrane oxygenation with an automatically driven blood pump on puppies. Artif Organs.

[11] Aissaoui N, Guerot E, Combes A, Delouche A, Chastre J, Leprince P, Leger P, Diehl JL, Fagon JY, Diebold B (2012). Two-dimensional strain rate and Doppler tissue myocardial velocities: analysis by echocardiography of hemodynamic and functional changes of the failed left ventricle during different degrees of extracorporeal life support. J Am Soc Echocardiogr.

[12] Burkhoff D, Sayer G, Doshi D, Uriel N (2015). Hemodynamics of mechanical circulatory support. J Am Coll Cardiol.

[13] Truby LK, Takeda K, Mauro C, Yuzefpolskaya M, Garan AR, Kirtane AJ, Topkara VK, Abrams D, Brodie D, Colombo PC, Naka Y, Takayama H (2017). Incidence and implications of left ventricular distention during venoarterial extracorporeal membrane oxygenation support. ASAIO J.

[14] Kato J, Seo T, Ando H, Takagi H, Ito T (1996). Coronary arterial perfusion during venoarterial extracorporeal membrane oxygenation. J Thorac Cardiovasc Surg.

[15] Fuhrman BP, Hernan LJ, Rotta AT, Heard CM, Rosenkranz ER (1999). Pathophysiology of cardiac extracorporeal membrane oxygenation. Artif Organs.

[16] Barbone A, Malvindi PG, Ferrara P, Tarelli G (2011). Left ventricle unloading by percutaneous pigtail during extracorporeal membrane oxygenation. Interact Cardiovasc Thorac Surg.

[17] Boulate D, Luyt CE, Pozzi M, Niculescu M, Combes A, Leprince P, Kirsch M (2013). Acute lung injury after mechanical circulatory support implantation in patients on extracorporeal life support: an unrecognized problem. Eur J Cardiothorac Surg.

[18] Soleimani B, Pae WE (2012). Management of left ventricular distension during peripheral extracorporeal membrane oxygenation for cardiogenic shock. Perfusion.

[19] Bavaria JE, Ratcliffe MB, Gupta KB, Wenger RK, Bogen DK, Edmunds LH (1988). Changes in left ventricular systolic wall stress during biventricular circulatory assistance. Ann Thorac Surg.

[20] Ostadal P, Mlcek M, Strunina S, Hrachovina M, Kruger A, Vondrakova D, Janotka M, Hala P, Kittnar O, Neuzil P (2016). Novel porcine model of acute severe cardiogenic shock developed by upper-body hypoxia. Physiol Res.

[21] Ostadal P, Kruger A, Vondrakova D, Janotka M, Psotova H, Neuzil P (2014). Noninvasive assessment of hemodynamic variables using near-infrared spectroscopy in patients experiencing cardiogenic shock and individuals undergoing venoarterial extracorporeal membrane oxygenation. J Crit Care.

[22] Shen I, Levy FH, Benak AM, Rothnie CL, O’Rourke PP, Duncan BW, Verrier ED (2001). Left ventricular dysfunction during extracorporeal membrane oxygenation in a hypoxemic swine model. Ann Thorac Surg.

[23] Tarzia V, Bortolussi G, Bianco R, Buratto E, Bejko J, Carrozzini M, De Franceschi M, Gregori D, Fichera D, Zanella F, Bottio T, Gerosa G (2015). Extracorporeal life support in cardiogenic shock: impact of acute versus chronic etiology on outcome. J Thorac Cardiovasc Surg.

[24] Howard RJ, Stopps TP, Moe GW, Gotlieb A, Armstrong PW (1988). Recovery from heart failure: structural and functional analysis in a canine model. Can J Physiol Pharmacol.

[25] Moe GW, Armstrong P (1999). Pacing-induced heart failure: a model to study the mechanism of disease progression and novel therapy in heart failure. Cardiovasc Res.

[26] Power JM, Tonkin AM (1999). Large animal models of heart failure. Aust N Z J Med.

[27] Schmitto JD, Mokashi SA, Lee LS, Popov AF, Coskun KO, Sossalla S, Sohns C, Bolman RM, Cohn LH, Chen FY (2011). Large animal models of chronic heart failure (CHF). J Surg Res.

[28] Gossage AM, Braxton Hicks JA (1913). On auricular fibrillation. Q J Med.

[29] Whipple GH, Sheffield LT, Woodman EG, Theophilis C, Friedman S (1962). Reversible congestive heart failure due to chronic rapid stimulation of the normal heart. Proc N Engl Cardiovasc Soci.

[30] Shinbane JS, Wood MA, Jensen DN, Ellenbogen KA, Fitzpatrick AP, Scheinman MM (1997). Tachycardia-induced cardiomyopathy: a review of animal models and clinical studies. J Am Coll Cardiol.

[31] Takagaki M, McCarthy PM, Tabata T, Dessoffy R, Cardon LA, Connor J, Ochiai Y, Thomas JD, Francis GS, Young JB, Fukamachi K (2002). Induction and maintenance of an experimental model of severe cardiomyopathy with a novel protocol of rapid ventricular pacing. J Thorac Cardiovasc Surg.

[32] Hála P, Mlček M, Ošťádal P, Janák D, Popková M, Bouček T, Lacko S, Kudlička J, Neužil P, Kittnar O (2018). Tachycardia-induced cardiomyopathy as a chronic heart failure model in swine. J Vis Exp.

[33] Gupta S, Figueredo VM (2014). Tachycardia mediated cardiomyopathy: pathophysiology, mechanisms, clinical features and management. Int J Cardiol.

[34] Nikolaidis LA, Hentosz T, Doverspike A, Huerbin R, Stolarski C, Shen YT, Shannon RP (2001). Mechanisms whereby rapid RV pacing causes LV dysfunction: perfusion-contraction matching and NO. Am J Physiol Heart Circ Physiol.

[35] Spinale FG, Hendrick DA, Crawford FA, Smith AC, Hamada Y, Carabello BA (1990). Chronic supraventricular tachycardia causes ventricular dysfunction and subendocardial injury in swine. Am J Physiol.

[36] Chow E, Woodard JC, Farrar DJ (1990). Rapid ventricular pacing in pigs: an experimental model of congestive heart failure. Am J Physiol.

[37] Hendrick DA, Smith AC, Kratz JM, Crawford FA, Spinale FG (1990). The pig as a model of tachycardia and dilated cardiomyopathy. Lab Anim Sci.

[38] Wyler F, Kaslin M, Hof R, Beglinger R, Becker M, Stalder G (1979). The Gottinger minipig as a laboratory animal. 5. Communication: cardiac output, its regional distribution and organ blood flow (author’s transl). Res Exp Med (Berl).

[39] Kass DA, Maughan WL, Guo ZM, Kono A, Sunagawa K, Sagawa K (1987). Comparative influence of load versus inotropic states on indexes of ventricular contractility: experimental and theoretical analysis based on pressure–volume relationships. Circulation.

[40] Glower DD, Spratt JA, Snow ND, Kabas JS, Davis JW, Olsen CO, Tyson GS, Sabiston DC, Rankin JS (1985). Linearity of the Frank-Starling relationship in the intact heart: the concept of preload recruitable stroke work. Circulation.

[41] Burkhoff D, Mirsky I, Suga H (2005). Assessment of systolic and diastolic ventricular properties via pressure–volume analysis: a guide for clinical, translational, and basic researchers. Am J Physiol Heart Circ Physiol.

[42] Walley KR (2016). Left ventricular function: time-varying elastance and left ventricular aortic coupling. Crit Care.

[43] Little WC (1985). The left ventricular dP/dt_max_-end-diastolic volume relation in closed-chest dogs. Circ Res.

[44] Bartlett RH, Gazzaniga AB, Huxtable RF, Schippers HC, O’Connor MJ, Jefferies MR (1977). Extracorporeal circulation (ECMO) in neonatal respiratory failure. J Thorac Cardiovasc Surg.

[45] Tanke RB, Daniels O, van Heijst AF, van Lier H, Festen C (2005). Cardiac dimensions during extracorporeal membrane oxygenation. Cardiol Young.

[46] Cheng R, Hachamovitch R, Kittleson M, Patel J, Arabia F, Moriguchi J, Esmailian F, Azarbal B (2014). Complications of extracorporeal membrane oxygenation for treatment of cardiogenic shock and cardiac arrest: a meta-analysis of 1,866 adult patients. Ann Thorac Surg.

[47] Kim S, Kim JS, Shin JS, Shin HJ (2019). How small is enough for the left heart decompression cannula during extracorporeal membrane oxygenation?. Acute Crit Care.

[48] Na SJ, Yang JH, Yang JH, Sung K, Choi JO, Hahn JY, Jeon ES, Cho YH (2019). Left heart decompression at venoarterial extracorporeal membrane oxygenation initiation in cardiogenic shock: prophylactic versus therapeutic strategy. J Thorac Dis.

[49] Ostadal P, Mlcek M, Gorhan H, Simundic I, Strunina S, Hrachovina M, Kruger A, Vondrakova D, Janotka M, Hala P, Mates M, Ostadal M, Leiter JC, Kittnar O, Neuzil P (2018). Electrocardiogram-synchronized pulsatile extracorporeal life support preserves left ventricular function and coronary flow in a porcine model of cardiogenic shock. PLoS ONE.

[50] Donker DW, Brodie D, Henriques JPS, Broome M (2019). Left ventricular unloading during veno-arterial ECMO: a review of percutaneous and surgical unloading interventions. Perfusion.

[51] Walther FJ, van de Bor M, Gangitano ES, Snyder JR (1990). Left and right ventricular output in newborn infants undergoing extracorporeal membrane oxygenation. Crit Care Med.

[52] Donker DW, Meuwese CL, Braithwaite SA, Broome M, van der Heijden JJ, Hermens JA, Platenkamp M, de Jong M, Janssen JGD, Balik M, Belohlavek J (2018). Echocardiography in extracorporeal life support: a key player in procedural guidance, tailoring and monitoring. Perfusion.

